# ADVANCED-COMFORT: TESTING A CARE PLANNING INTERVENTION FOR NURSING HOME RESIDENTS WITH ADVANCED DEMENTIA

**DOI:** 10.1093/geroni/igad104.1859

**Published:** 2023-12-21

**Authors:** Ruth Palan Lopez, Andrea Wei, Jenna Locke, Evan Plys

**Affiliations:** MGH Institute of Health Professions, Sharon, Massachusetts, United States; MGH Institute of Health Professions, Boston, Massachusetts, United States; MGH Institute of Health Professions, Boston, Massachusetts, United States; Massachusetts General Hospital/ Harvard Medical School, Boston, Massachusetts, United States

## Abstract

Many nursing home (NH) residents with advanced dementia receive burdensome interventions rather than interventions that promote comfort or quality of life. The purpose of this study was to test the usability of a novel intervention called ADVANCED-Comfort that aims to enhance the provision of personalized, comfort focused care for residents with advanced dementia. The intervention consisted of structured care plan meetings between the NH team and proxies of residents with dementia (e.g., family members). Using the ADVANCED-Comfort workbook, proxies and staff created individualized care plans addressing 6 domains adapted from the Age Friendly Health System Framework (what Matters; Meaningful activities; Mealtime; Mobility; Medications and treatments; and Make comfortable). We evaluated the intervention using surveys, observation, and exit interviews. We found the intervention to be highly usable (acceptable, appropriate, and feasible) and exit interviews identified several promising outcomes. Both families and staff reported that the intervention improved communication and trust. Families reported that they had a newfound trust in staff to provide consistent quality care. Staff reported that the intervention helped them build better relationships with families and allowed families to articulate what they prefer rather than waiting until “something goes wrong.” Staff also reported that the intervention improved their excitement to go to work and enhanced their sense of teamwork. Finally, both staff and proxies reported exciting implications for residents such as a reduction in antipsychotic medication, decrease in distressed behaviors, weight stabilization, and initiation of hospice. Based on these preliminary findings, additional testing of the ADVANCED-Comfort intervention is warranted.

